# The Use of Heparin during Endovascular Peripheral Arterial Interventions: A Synopsis

**DOI:** 10.1155/2016/1456298

**Published:** 2016-04-17

**Authors:** Arno M. Wiersema, Christopher Watts, Alexandra C. Durran, Michel M. P. J. Reijnen, Otto M. van Delden, Frans L. Moll, Jan Albert Vos

**Affiliations:** ^1^Department of Surgery, Division of Vascular Surgery, Westfriesgasthuis, Maelsonstraat 3, 1624 NP Hoorn, Netherlands; ^2^Department of Surgery, Division of Vascular Surgery, University Medical Centre Utrecht, University of Utrecht, Postbus 85500, 3508 GA Utrecht, Netherlands; ^3^Department of Radiology, Salisbury District Hospital, Odstock Road, Salisbury, Wiltshire SP2 8BJ, UK; ^4^Department of Radiology, Peninsula Radiology Academy, Plymouth PL6 5WR, UK; ^5^Department of Surgery, Rijnstate Hospital, Arnhem, Postbus 9555, 6800 TA Arnhem, Netherlands; ^6^Department of Radiology, Division of Interventional Radiology, Academic Medical Centre, University of Amsterdam, Postbus 22660, 1100 DD Amsterdam, Netherlands; ^7^Department of Radiology, Division of Interventional Radiology, St. Antonius Hospital, Postbus 2500, 3430 EM Nieuwegein, Netherlands

## Abstract

A large variety exists for many aspects of the use of heparin as periprocedural prophylactic antithrombotics (PPAT) during peripheral arterial interventions (PAI). This variation is present, not only within countries, but also between them. Due to a lack of (robust) data, no systematic review on the use of heparin during PAI could be justified. A synopsis of all available literature on heparin during PAI describes that heparin is used on technical equipment to reduce the thrombogenicity and in the flushing solution with saline. Heparin could have a cumulative anticoagulant effect when used in combination with ionic contrast medium. No level-1 evidence exists on the use of heparin. A measurement of actual anticoagulation status by means of an activated clotting time should be mandatory.

## 1. Introduction

Recent extensive surveys amongst interventional radiologists (IR) have shown that (unfractionated) heparin is used by almost all European IR during peripheral arterial interventions (PAI) [1, 2]. PAI are defined as all noncardiac and noncerebral arterial interventions. Heparin is used as a periprocedural prophylactic antithrombotic (PPAT) agent to prevent distal and proximal arterial thromboembolic complications (ATEC) and to reduce the formation of thrombus on catheters and to prevent the formation of blood clots within catheters. Heparin is also used during PAI as a flushing solution on the sideport of a sheath, mostly diluted with saline (hepsal), and to coat catheters and wires. This current widespread use of heparin is in accordance with earlier reports from Europe and the United States [3–5].

The harmful side effects of heparin are also well recognized: a higher bleeding tendency, resulting in local and systemic bleeding complications. It is self-evident that all bleeding complications enhanced or caused by heparin have a negative influence on results of PAI.

The use of heparin may also result in heparin-induced-thrombocytopenia (HIT), a rare but possibly limb or life-threatening complication [6].

From literature it is known that heparin has no linear dose-response curve and elimination curve in the vascular patient [7, 8]. This underscores the necessity of measuring the actual, clinical effect of heparin either by checking the activated clotting time (ACT) or by performing a heparin concentration or dose-response test [9, 10]. Surveys from the United Kingdom (UK) and Netherlands showed that only a small minority of IR implemented such a point-of-care measurement in daily routine [1, 2]. This policy of not performing a measurement of the actual heparin-effect adds to the risk of thromboembolic complications, due to insufficient dosing of heparin and of bleeding complications, due to “over-dosing” of heparin, during PAI.

The incidence of the complications caused by the use of heparin in PAI is probably underestimated, as a majority of interventional radiology departments around the world still do not apply a strict complication registry and until now, no centralized complication registration is mandatory for IR. In most Dutch and UK hospitals the vascular patients for peripheral arterial interventions are admitted on a vascular surgery ward by a vascular surgeon, who also performs the follow-up of the patients after those interventions. Consequently, any late complications are probably registered by the vascular surgeon and not by the IR. Additionally, it has been stipulated that a general underregistration of complications by medical specialists exists [11].

Current guidelines in IR, such as TASC II [12] and CIRSE [13], advise the use of heparin as periprocedural prophylactic antithrombotic. But despite these guidelines and the worldwide use of heparin for the past 30-plus years, there still is no consensus on many aspects of its use in PAI. Mentioned surveys in the UK and Netherlands [1, 2, 5] showed a significant variation in all aspects of heparin use during PAI. Alarmingly, the described variation in Netherlands and the UK was not only present in both countries, but also different between those countries. This emphasises the need for new, practical level-1 evidence based guidelines.

For the purpose of creating such guidelines, a study group was formed in Netherlands. This group was instituted in close collaboration between the Dutch Society of Vascular Surgery and the Dutch Society of Interventional Radiology (NGIR) and was named CAPPA: Consensus on Arterial Periprocedural Anticoagulation [2, 14–16]. Collaboration was established between authors from the UK survey and the Dutch survey and results of those combined data were incorporated in a recent publication [2].

To objectively assess the results of both surveys from Netherlands and the UK, we intended to perform a systematic review on the intraprocedural use of heparin or other antithrombotics. After a literature search and an attempt to execute a review according to PRISMA guidelines, it appeared that no systematic review could be justified due to the lack of randomised data.

Therefore we decided to perform an in-depth analysis of all available literature on heparin or other PPAT in IR.

## 2. Heparin: Pharmacokinetics and Mechanism of Action

Heparin is a glycosaminoglycan and influences the coagulation cascade mainly through an interaction with antithrombin III (AT-III). This combination of enzyme and inhibitor inactivates coagulation enzymes, mainly thrombin (IIa) and Xa. Heparin is heterogeneous in its size and weight of molecules, its effect on coagulation, and its pharmacokinetic effects. These facts explain why heparin has a nonlinear effect on coagulation. The higher molecular weight molecules of heparin are subject to a faster biological clearance from the blood. This faster clearance also causes accumulation of lower molecular weight molecules in vivo. These molecules, however, exhibit low activity on AT-III and are therefore of less clinical influence on coagulation. In addition heparin binds nonspecifically to proteins and cells in the blood of the patient. This causes a further limitation on the clinical effect of heparin administration. This nonspecific binding causes low bioavailability at low doses of heparin and a short plasma half-life and creates a large variability in anticoagulant effect amongst patients with vascular disease [17].

## 3. Heparin in Flushing Solution and as Bolus

Heparin is used during arterial angiography, both as a bolus and in the flushing solution. It presumably reduces thrombotic complications by reduction of thrombus formation on wires, sheaths, and catheters and by reducing the effects of the hypercoagulable state present in vascular patients [18].

### 3.1. Historical Data

During the early days of angiography [19], periprocedural anticoagulation received considerable attention. Because of its rapidly adopted standardized use, most publications on heparin in IR are from the 1970s and 80s. Heparin as routinely used antithrombotic was advocated by Wallace et al. in 1972 [20]. When heparin is used as a bolus instead of only in the flushing solution, significant fewer thrombotic complications were present, while no increase in hemorrhagic complications occurred [20, 21]. It was also shown that heparin used as a bolus resulted in immediate effective anticoagulation, while heparin in the flushing solution resulted in maximal anticoagulation effect at the end of the procedure. Another study [22] indicated that bolus injection of heparin provided a better anticoagulation effect than continuous infusion. Since the 1990s [3], all these publications resulted in widely accepted and advocated use of heparin as PPAT in arterial interventions. Recommendations included the administration of 2000–3000 IU intra-arterially or intravenously as bolus and 2000 IU/L hepsal as flushing solution.

### 3.2. Current Data

More recently, only a limited number of studies have been published on heparin as PPAT and on the comparison of heparin with new anticoagulants. In 2002 a study was performed [23] on coronary and peripheral interventions in which heparin was replaced as antithrombotic by a low molecular weight heparin (LMWH), enoxaparin, and was combined with a glycoprotein IIb/IIIa receptor antagonist (eptifibatide). No robust conclusions could be made on whether this combination provided better results than heparin alone, amongst others because only a small number of arterial interventions (*n* = 21) were included. Sheikh et al. published in 2009 [24] that no ACC/AHA guidelines existed on the use of heparin as PPAT and that most anticoagulation strategies in PAI were directly extrapolated from studies performed during coronary interventions.

Studies during coronary interventions indicated that bivalirudin has the same efficacy as heparin as PPAT, but with less ischemic and bleeding complications [25]. No clinically relevant differences were found in a study comparing heparin with bivalirudin during PAI and heparin was considerably less expensive than bivalirudin (US $6 versus 547 per procedure) [24]. The same conclusions could be drawn from a study comparing heparin and bivalirudin during EVAR ([15] and [26]). Both studies [24] and [48] concluded that RCTs are needed to further evaluate the possible advantages of direct thrombin inhibitors over heparin during PAI. Until today, no results of such trials have been published.

Another alternative for heparin is the low molecular weight heparins (LMWH). Compared to heparin, LMWH does not enhance platelet aggregation, is less sensitive to neutralisation by activated platelets, and demonstrates a higher antithrombotic activity, higher bioavailability, and longer half-life than heparin. Also heparin-induced-thrombocytopenia caused by LMWH is less frequent [17]. As with other prophylactic antithrombotics, the use of LMWH versus heparin has been well established for coronary interventions. LMWH proved to reduce major bleeding complications, while not increasing ischemic study endpoints [27, 28]. Duschek et al. [29] performed a RCT using LMWH or heparin during PAI. This is the only RCT on the use of 2 different periprocedural prophylactic antithrombotics during peripheral arterial interventions that could be retrieved from literature. In this study the primary composed endpoints were better for enoxaparin than for heparin. Endpoints were defined as clinical performance of enoxaparin by comparing the peri-interventional rate of thromboembolic occlusion (efficacy) of endovascular reconstructed areas, of bleeding complications, and of any necessary reintervention for any PTA related bleeding, 10.5% versus 2.5% and *P* < 0.05. The concomitant use of acetyl-salicylic-acid (ASA) increased the incidence of bleeding complications in the heparin group, but not in the enoxaparin group. In 2012, a retrospective evaluation of using heparin or no heparin during peripheral interventions was published [30]. Results were described for 220 arterial procedures with the use of a bolus of heparin and 110 in which no bolus of heparin was administered. Although applied doses varied, a dose of 5000 IU was used predominantly. All procedures were performed with a hepsal flushing solution with a concentration of 1000 IU per 500 mL. This study showed an increased risk for bleeding complications in the heparin group at the access site (OR = 5.7; 95% CI = 1.3–25) without a reduction of arterial thromboembolic complications in the heparin group. The use of protamine or no protamine did not result in any differences in complications and no monitoring of anticoagulation using the ACT was performed during the included procedures. The authors stressed the absence of level-1 data to support the use of heparin as PPAT during peripheral arterial interventions and concluded that RCTs should be started.

## 4. Heparin and Contrast Medium

Although taken for granted nowadays by most IR, the type of contrast medium and its influence on clotting and arterial thromboembolic complications have been the subject of considerable discussion.

### 4.1. Historical Data

Already in 1896, almost within a year of the introduction of X-ray by Röntgen, contrast medium was used [31]. These contrast agents were all ionic, until stable nonionic monomers were developed in the late 1960s. It took until 1985 when nonionic contrast medium (NICM) was introduced for use in angiography [31]. Both ionic low osmolar contrast medium (ICM) and nonionic isoosmolar contrast medium (NICM) display pro- or antithrombotic properties. Clot formation could be inhibited by ionic contrast medium (ioxaglate), but only if it is present in blood in more than 8% concentration. Despite dilution when the contrast medium mixes with blood, it is highly probable that a concentration of 8% of ionic contrast medium is reached. For nonionic contrast this threshold of displaying inhibition of clot formation is a 30% concentration. So therefore it is likely that ionic contrast medium reduces clot formation more than nonionic [4, 32–34].

### 4.2. Current Data

A reduction in the thrombogenicity during angiography by thrombus formation in and at the angiographic catheter could be achieved by the additional administration of heparin. The anticoagulant effect of ionic contrast and heparin is cumulative and thereby increase the active anticoagulation period from 4 hours with systemic heparin alone to 6 hours with the combination of heparin and ionic contrast medium [35, 36].

## 5. Heparin and Guide Wires, Sheaths, and Catheters

Guide wires, sheaths, and catheters can play an important role in angiography related arterial thromboembolic complications. Clots may form on the outside surface of these devices and thrombus can also be encased inside the lumen and then be pushed into the circulation when wires or other devices are inserted through that lumen. In addition, the injection of contrast medium through the lumen can cause dispersing of thrombus material. Another pathway of thrombotic complications caused by wires, sheaths, and catheters is when they are removed from the puncture site. Formed clots, which are adherent to the devices, can be stripped off and thrombus is thereby released in the arterial circulation distal to the puncture site. Since the introduction of angiography, focus has been directed to reducing this thrombogenicity of wires, sheaths, and catheters.

### 5.1. Historical Data

It was shown that clot formation could be detected on all catheters when these were positioned inside a blood vessel [18, 19, 37]. In the 1970s heparin coated catheters were introduced in clinical practice [21, 22]. These would reduce but probably not eliminate (post)catheterization thrombosis. At first the heparin was washed rapidly from the catheters when in contact with blood.

### 5.2. Current Data

From the 1980s, the heparin was stabilized and not washed off within the hour when in contact with blood. This “striking reduction of thrombogenicity achieved with heparinization” was later confirmed by several other studies [23, 24].

To even further reduce thrombogenicity of wires, sheaths, and catheters, a hydrophilic coating was introduced. The combination of heparin and hydrophilic coating proved to be highly nonthrombogenic [23, 38, 39].

## 6. Measuring the Clinical Effect of Heparin

### 6.1. Historical Data

As mentioned earlier, heparin as PPAT during peripheral arterial interventions was introduced mainly by extrapolation from coronary interventions. The use of a bolus of heparin and its dosage was adopted and implemented as “standard of care” in IR. Surprisingly though, the standardized use of performing a reliable measurement of the actual effect of heparin on coagulation status was not directly extrapolated and implemented in daily use from coronary to peripheral interventions. Although the heparin dose used during coronary interventions is, on average, larger than that used during peripheral interventions, this difference in the amount of administered heparin does not explain and justify the complete absence of monitoring the actual anticoagulation during peripheral arterial interventions. Every now and then focus is directed towards this topic of measuring the actual effect of heparin during PAI. As was convincingly proven during coronary interventions the activated clotting time (ACT) correlates better with the effect of heparin than the previously used activated partial thromboplastin time (APTT) [40–44].

In 1996, an editorial by Klein and Agarwal [45] showed that some controversies on ACT measurement during PCI were present at that time. These controversies apparently still exist and can also be applied to PAI:

 What should the optimal therapeutic goal of the ACT be?
 Measured values of ACT differ between used devices and procedures.
 At what time during the procedure should the ACT be monitored?

At the start of the 21st century, it was advocated that, during and after PAI, monitoring of anticoagulation is a “crucial responsibility.” It was stipulated that data should be gathered as soon as possible, on how to monitor anticoagulation and what actions to take at different values of ACT during PAI [45]. Jackson and Dawson [4] stated the following in their 1995 inventory of angiographic practice in the UK: “… radiologists should be prepared to follow cardiologists and invest in ACT meters if heparinization is to be more than folklore in the important area of angioplasty.”

### 6.2. Current Data

It took until 2010 before a large cohort of PAI patients (*n* = 4743) was described [42] in which a correlation was sought between heparin dosage, measured ACT, and the optimal degree of anticoagulation related to clinical parameters. Kasapis et al. [42] stated in that publication that, at that moment in time (2010), the optimal heparin anticoagulation during PAI was unknown and that recommendations were based on coronary literature and empirical guidelines, not based on any robust evidence for PAI. In those guidelines a target ACT of 200–250 seconds was advocated. They further evaluated the applied heparin dose in 4743 patients (<60 IU/kg or >60 IU/kg) and the ACT, which was obtained in 1246 patients (peak value < 250 seconds or > 250 seconds). They related bleeding complications with the depicted cut-off values. Main conclusions from this large registry were that a higher total heparin dose (>60 IU/kg) and a peak procedural ACT of >250 seconds were strong predictors of significantly increased postprocedural bleeding events. The technical and procedural success was high and did not differ between the described groups with higher or lower heparin dose or peak ACT. Deduced from these results, it was strongly suggested that, during PAI, a body-weight-dependent dose of up to 60 IU/kg should be administered, while the ACT should have a target peak value of <250 seconds.

## 7. Protamine

To reduce the higher bleeding tendency caused by heparin administration, protamine sulphate has been used to reverse the effect of heparin. Protamine is a heterogeneous mixture of highly cationic polypeptides, originally purified from salmon sperm, but nowadays produced through recombinant biotechnology. Protamine has been subject of much controversy. It can cause adverse and potentially life-threatening complications such as a severe allergic reaction, systemic arterial hypotension, decreased cardiac output, decreased oxygen consumption, bradycardia, and even death [46]. Additionally, when protamine is not bound to heparin in blood, it expresses anticoagulant properties, thereby creating a contradictive effect in the vascular patient when the dose of protamine is not exactly matched with the circulating heparin at that precise moment. In Netherlands the use of protamine by interventional radiologists is incidental (<1%) [2], but in the United States of America (USA) and the United Kingdom, protamine was used more regularly [1, 3]. Considering the fact that only a small minority of interventional radiologists measure the actual, clinical effect of heparin in the patient and the fact that heparin has no linear dose-response curve and elimination curve, standardized reversal of heparin with protamine seems, at least, not evidence based.

## 8. Discussion

Heparin is used by almost all interventionalists, being interventional radiologists or vascular surgeons, around the world as periprocedural prophylactic antithrombotic during peripheral arterial interventions. Heparin is also used on all disposables to reduce the thrombogenicity of those materials. Additionally, heparin is used in the flushing solution with saline and heparin has a cumulative anticoagulant effect when used in combination with ionic contrast medium. No level-1 evidence exists on the use of heparin as a bolus as PPAT during peripheral arterial interventions and a wide variation between institutions and between countries exists on all aspects of the use of heparin during these interventions. Only a small minority of IR or vascular surgeons use a measurement of actual anticoagulation by means of an ACT, when using heparin. The use of a bolus of heparin influences obtained results during PAI. It could result in more thromboembolic complications or bleeding complications, especially when no measurement of actual (anti)coagulation status is performed.

The bolus use of heparin has been introduced in endovascular interventions mainly by direct extrapolation from coronary interventions without RCTs on its use in peripheral interventions. Reasons why these results and protocols from PCI should be extrapolated with caution and should be tested in trials for periprocedural use in endovascular peripheral arterial interventions are numerous and subject to discussion: catheters, wires, and other devices used during coronary interventions are different from those used in PAI and the target vessels differ in size and probably the histopathological responses to balloon- and stent-dilatation and local drug deliverance by balloon or stent. Also the hemodynamics in the coronary system might be different from that in the aortoiliac and infrainguinal arterial vessels. The myocardium is a different muscle system than that in the leg. Furthermore the risk-benefit ratio in the heart is completely different from that in the leg and the reserve and capacity to regenerate of muscles in the leg are far more extensive than those of the myocardium. Because of this, a higher target anticoagulation level is deemed necessary and a higher percentage of bleeding complications can be accepted in coronary intervention at the benefit of less myocardial infarctions and possible deaths.

Despite technical developments, the key to preventing thromboembolic complications is still sound technical handling of wires, sheaths, and catheters during endovascular procedures. Careful positioning, gentle handling, using as little contrast medium as possible, and reducing the contact of materials with the vessel wall to an absolute minimum are, amongst others, self-evident but need to be continuously emphasised and employed.

The details of the administration of the flushing solution through a sideport are important. If, for example, continuous low pressure flushing is applied when using a pigtail catheter, or other catheters with end and side holes, thrombus may form in the distal part of these catheters. This thrombus formation will only be sufficiently prevented when intermittent rapid high pressure flushing is applied [37].

Although the use of heparin as PPAT in peripheral interventions was extrapolated from coronary interventions, the use of a measurement of actual anticoagulation has not been widely incorporated as standard of care during PAI by interventional radiologists or vascular surgeons. It has been shown that such a measurement is essential to tailor the anticoagulant therapy in the individual patient. Heparin has been shown to have a nonlinear response curve and a nonlinear elimination pattern in the vascular patient. This results in an unpredictable influence on coagulation in the individual patient undergoing PAI, resulting in preventable either thromboembolic or bleeding complications. Measurement of the ACT with a point-of-care device during peripheral arterial interventions should become one of the foundations of interventions in the arterial system.

In conclusion, in the current era of evidence based medicine, the use of heparin as periprocedural prophylactic antithrombotic during peripheral arterial interventions needs to be evaluated by means of RCTs. Performing a measurement of actual anticoagulation status when performing PAI with a periprocedural prophylactic antithrombotic should be adopted as mandatory by arterial interventionalists (IR and vascular surgeons) around the world to prevent unnecessary risks for the vascular patient. The CAPPA group from Netherlands will institute such trials, hopefully in close collaboration with other countries, to establish the role of a bolus of heparin as PPAT. Once the beneficiary or harmful effect of this use of heparin has been established, further RCTs can be designed to evaluate the role of the new anticoagulants, such as the direct thrombin inhibitors, during peripheral arterial interventions.
